# Transsexual men’s experiences of childbirth and postpartum in the light of transcultural care *

**DOI:** 10.1590/1518-8345.7040.4212

**Published:** 2024-11-22

**Authors:** Danilo Martins Roque Pereira, Ednaldo Cavalcante de Araújo, Sheyla Costa de Oliveira, Anderson Reis de Sousa, Mariana Mercês Mesquita Espíndola, Dante Eduardo Barbosa de Lemos

**Affiliations:** ^1^ Universidade Federal de Pernambuco, Recife, PE, Brazil.; ^2^ Scholarship holder at the Coordenação de Aperfeiçoamento de Pessoal de Nível Superior (CAPES), Brazil.; ^3^ Universidade Federal da Bahia, Salvador, BA, Brazil.; ^4^ Universidade Federal de Pernambuco, Departamento de Enfermagem, Recife, PE, Brazil.

**Keywords:** Transgender Persons, Parturition, Men’s Health, Primary Health Care, Sexual and Gender Minorities, Nursing

## Abstract

to unveil the experiences of transsexual men during childbirth and postpartum in the light of the Theory of Diversity and Universality of Cultural Care.

a qualitative and descriptive study using the multiple case study method. Data was collected using an intentional sample of five transsexual men, selected on the basis of convenience and availability. The interviews were transcribed in full and the results were organized and adapted to the Sunrise Model.

the majority of the participants were «primiparous» and had given birth by cesarean section. In adapting the Sunrise Model, we observed the encouragement of medicalization and mechanistic management of childbirth; fear of natural childbirth; violence perpetrated against transsexual men resulting from the difficulty of access to information by the pregnant man, and obstetric care not qualified to meet the needs of the public, resulting in fragile care, with dissatisfaction with the health service.

the experiences of transsexual men during childbirth and the postpartum period are a mixture of experiences that generate damage, especially when linked to situations of transphobic violence and violation of rights

## Highlights

**(1)** Provides an opportunity to reflect on obstetric care for transsexual men.

**(2)** Influence of social stigma about normal childbirth on the choice of delivery route.

**(3)** Need for hospital care that recognizes the uniqueness of the parturient.

**(4)** The environmental context can reveal more negative situations in care.

**(5)** Care is enhanced by coping strategies.

## Introduction

 From the point of view of comprehensive health, the process of pregnancy in transsexual men can result in positive and negative experiences, since most of them have preserved the organs of the reproductive system, such as the vagina, uterus, fallopian tubes, and ovaries, with the ability to gestate and give birth, making gynecological and obstetric health care essential for them, as a guarantee of sexual and reproductive rights, safety and pregnancy-puerperal protection ^(^ 1 ^)^ . 

 It is known that in Australia, between 2013 and 2018, a total of 205 trans men gave birth, while in Brazil, trans and non-binary people who become pregnant are not identified in health service data due to the impossibility of filling in the «gender identity» question in places of reference for obstetric care, such as birth centers or maternity hospitals, which makes it impossible to analyze the different experiences during childbirth and the puerperium, as well as making visible the inequalities in access and the effects on individual integral health ^(^ 2 ^)^ . 

In particular, there is a significant gap in nursing in relation to health care for transsexual men during the pregnancy-puerperal cycle, since this care is discussed during professional training from a cis-heteronormative perspective, contributing to a limited view of care, excluding the specific experiences and needs of this social group. In this way, this research could support advanced nursing practice in order to consider the sociocultural particularities of this population segment during childbirth and the immediate postpartum period.

 It should be noted that transsexual men often express discomfort with their own bodies and/or genitalia to health professionals during pregnancy and that they feel uncomfortable with everyday health care situations, such as gynecological exams and vaginal examinations from the 36th week of pregnancy onwards. In addition, they assume that these tests can be carried out without their consent, including during labor, which contributes to increased fear and anxiety and not seeking health services ^(^ 3 ^-^ 4 ^)^ . In this respect, it should be noted that these tests help to identify health problems and even prevent a possible premature birth, as they also aim to analyze the pelvis in order to assess the structure of the vagina and cervix. 

 In this context, Madeleine Leininger’s Theory of Diversity and Universality of Cultural Care (TDUCC), in its conceptual model, Sunrise, proposed considering care for human beings in a way that is congruent with knowledge of their culture and worldview, discovering the meaning of transcultural care, based on practices specifically geared to each culture and its influencing factors in the provision of health care. The theorist interprets the existence of cultural diversity and universality as a focus in structuring care so that the person can be assisted in a satisfactory and humanistic way, and the concepts of “Culture”, “Worldview”, “Environmental context”, “Care” and “Health” are fundamental to understanding this study ^(^ 5 ^-^ 6 ^)^ . 

 It is important to reduce inequalities and disparities in health, promote equity in care, and make it possible to guarantee human rights and social justice in order to achieve the Sustainable Development Goals ^(^ 7 ^-^ 8 ^)^ . In this sense, this study makes it possible to reflect on obstetric care for transgender men during childbirth and the postpartum period in order to promote significant changes in work processes in different public and private professional settings. In view of the above, the following guiding question was formulated: what are the experiences of transsexual men during childbirth and postpartum? To this end, the aim was to unveil the experiences of transsexual men during childbirth and postpartum in the light of the Theory of Diversity and Universality of Cultural Care. 

## Method

### Study design and location

 This is a qualitative study ^(^ 9 ^-^ 10 ^)^ conducted using the Multiple Case Study method ^(^ 11 ^)^ . The setting investigated is linked to the State Health Department, through the State Coordination of Comprehensive Care for Lesbian, Gay, Bisexual, Transvestite, and Transgender (LGBT) Health in Pernambuco (NP), and at a national level with people linked to the National LGBT Alliance. 

### Sample definition

 Five “parturient”, transsexual men took part in the study. The sample was intentional ^(^ 12 ^)^ , made up of participants selected on the basis of convenience and availability, after establishing the following inclusion criteria: being a transsexual man with experience of vaginal labor or cesarean section; being 18 years old or over. The exclusion criteria were: transsexual men who had experienced labor and who did not have technological resources such as a computer, cell phone, or Internet, among others, to enable the interview to take place. Participants were included by progressive insertion based on the theoretical saturation of the responses to the semi-structured interviews ^(^ 13 ^)^ . 

### Data collection

 Data was collected in September and October 2021. For this purpose, the technique of individual interviews ^(^ 13 ^)^ was used, in virtual (remote) mode, through the Google Meet ^®^ platform, in a single meeting, with a semi-structured script, composed of closed questions related to sociodemographic characteristics and open questions related to the empirical phenomenon investigated, namely: 1. Tell me about your experience during childbirth and the postpartum period; 2. Tell me about the health care provided by the health team during childbirth and the postpartum period; 3. If you chose to breastfeed in the postpartum period, tell me about your experience; 4. Do you know any other transsexual men who have become pregnant or are pregnant? Tell me about your experiences. The sociodemographic characterization of the participants was carried out after a questionnaire was made available before the interviews. Participants were recruited through contact with partner institutions, which took place on the main digital social networks accessed in Brazil: Facebook ^®^ , Instagram ^®^ and WhatsApp® ^(^ 14 ^)^ . 

 The consent forms for the Free and Informed Consent Term (FICT) and the Image and Testimonial Authorization Form were made available for signing via the Google Forms ^®^ platform (online). For each interview, they were asked to turn on their cell phone or computer camera to capture images, body language, and voice intonations via the Google Meet ^®^ platform, taking an average of one hour for each interview. 

 The interviews were carried out by the main researcher, who is a member of the LGBT community and has expertise in carrying out qualitative studies, which contributed to the process of collecting the information. They were then transcribed in full and made available to the participants so that the content could be validated, making adjustments and corrections if necessary. At this stage, all the participants read the material and no changes to the content were suggested. All the material was subjected to classic lexicographic analysis using the Reinert Method ^(^ 15 ^)^ for classifying text segments, instrumented by the free software *Interface de R pour les Analyses Multidimensionnelles de Textes et de Questionnaires* (IRAMuTeQ) version 7.0 ^(^ 16 ^)^ . Using IRAMuTeQ, it was possible to categorize the data by assessing similarity ^(^ 17 ^-^ 19 ^)^ . 

### Data processing and analysis

 The central findings of the Multiple Case Study were discussed theoretically based on the assumptions of the TDUCC ^(^ 20 ^)^ . The analysis of the data was anchored in the TDUCC, proposed by Madeleine Leininger, which addresses the need for the professional to consider the cultural context in which the individual is inserted, providing harmonious care to that local identity, with five concepts that support it being listed, namely culture, worldview, environmental context, care, and health; associated with the scientific literature pertinent to the subject ^(^ 20 ^)^ . 

### Ethical aspects

 This study was approved by the Research Ethics Committee of the Federal University of Pernambuco (UFPE), under CAAE No. 47777421.0.0000.5208 and opinion number 4.862.503/2021, following the recommendations of Resolution No. 466 of December 12, 2012 of the Brazilian National Health Council ^(^ 21 ^)^ . 

## Results

**Figure 1.**
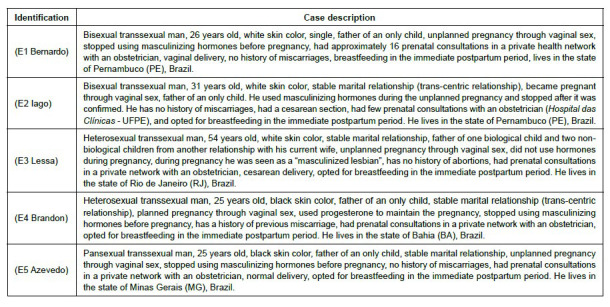
Multiple cases of transsexual men participating in the study (n = 5). Recife, PE, Brazil, 2023

Figure 1 shows the multiple cases of the «parturient» transsexual men in the study, identified by self-declared pseudonyms at the time of the interviews, according to their pregnancy-parturition profile. 

**Figure 2.**
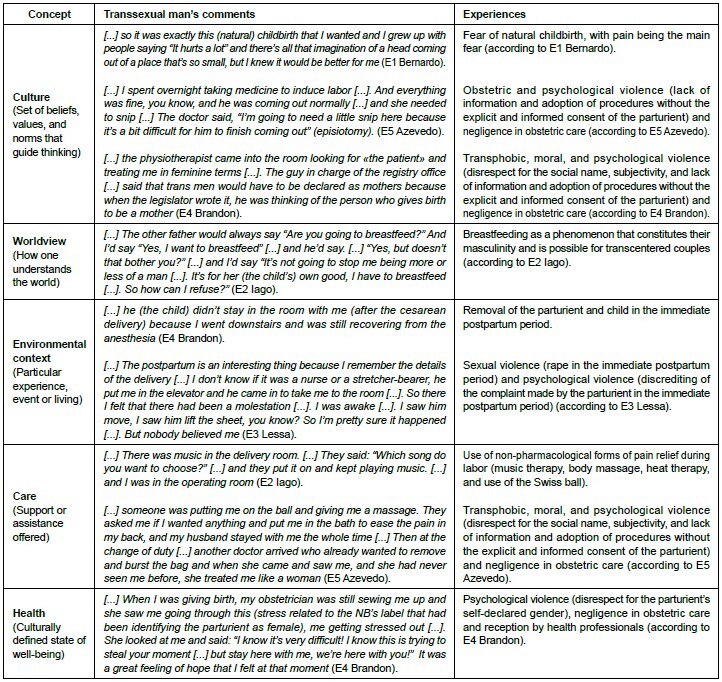
Experiences attributed to childbirth and immediate postpartum by transsexual men adapted to the Sunrise Model. Recife, PE, Brazil, 2023

Figure 2 shows the grouping of the most significant records by concept, based on the adaptation of the Sunrise Model, proposed by the TDUCC, allocating the statements in line with the experiences of transsexual men during childbirth and postpartum. Thus, the reports expose the fear of childbirth, obstetric violence, transphobic violence, moral violence, psychological violence, sexual violence, or violence due to negligence, among others, as shown below. 

**Figure 3.**
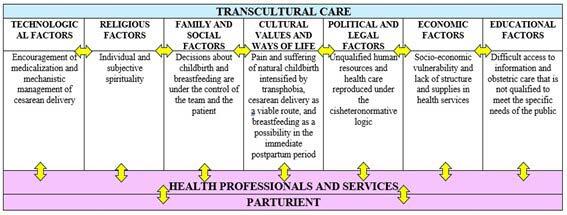
Adaptation of the Sunrise Model. Values that influence health care for parturients. Recife, PE, Brazil, 2023

**Figure 4.**
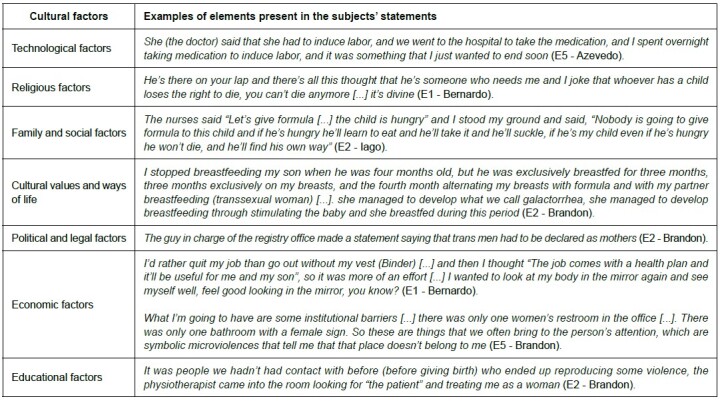
Cultural factors that influence health care and the elements present in the speech of parturients. Recife, PE, Brazil, 2023

Figure 3 shows the result of constructing the adaptation of the Sunrise Model, translated into key ideas that recur most frequently in the speeches of transsexual men who give birth, highlighting the intrinsic aspects of childbirth and postpartum experienced by these individuals, which need to be considered in order to recognize transcultural care. Figure 4 shows the relationship between cultural factors and the elements present in the speeches of the individuals participating in the study. 

## Discussion

The lack of national and international scientific literature addressing the specificities of transsexual men during pregnancy, childbirth, and the puerperium is noteworthy, reaffirming the need for this study, which encompasses Obstetrics and Nursing in discussions about the relationships between health, gender, and sexualities. In view of the above, this study presents relevant contributions and implications in these areas and in the advancement of scientific knowledge in prenatal and obstetric care for different gender identities, especially with regard to the visibility of the experiences of transsexual men during childbirth and the immediate postpartum period, under the analysis of the concepts of “Culture”, “Worldview”, “Environmental Context”, “Care” and “Health” proposed by Madeleine Leininger’s TDUCC.

 In the item referring to «culture», we recorded the perception that transsexual men had of natural childbirth and the acts of violence that occurred at this time, which were sometimes naturalized. In this sense, the social stigma that culturally considers normal childbirth to be dangerous, which generates pain and suffering, has a significant influence on the experiences of transsexual men. This corroborates what Leininger said, that culture can guide an individual or group in their actions and shape the way they would like to be cared for. Part of this feeling is related to the health care offered by the Unified Health System (UHS), which is often seen as unqualified since they believe that care is provided after long waiting times, and this justifies the choice of cesarean section surgery even when there are conditions for natural childbirth, relating the surgical route as quick and safe ^(^ 22 ^)^ . 

 With the medicalization of childbirth, it has become socially widespread that the effects of medicines and technical and technological procedures carried out by health professionals are more efficient than the body’s own physiology. Scientific evidence shows that the main factors that trigger fear of natural childbirth among transsexual men are reports of pain from family and friends who have been through this moment, anxiety related to suffering during normal childbirth, insecurity about not being able to complete the birth, and fear that it will cause complications for the newborn (NB) ^(^ 23 ^)^ . The fear of this moment may also be related to changes in the vaginal canal (the wall of the vagina consists of three layers: mucosa, muscle, and adventitia), due to the use of testosterone during the gender affirmation process and concerns about insufficient care, such as the lack of resources in health services, or regarding the cross-cultural competence of professionals in assisting the delivery of a transsexual man, given the incipiency of this discussion in health training courses ^(^ 3 ^-^ 4 ^,^ 24 ^)^ . 

 In one of the study participants’ experiences, in which “the parturient” opted for the normal delivery route, there was the procedure of induction of labor and episiotomy. There was a lack of information on the part of the user about what the procedure was and a decision was made to carry out the procedures without the “parturient’s” explicit and informed consent, which could also be characterized as obstetric violence. It should be noted that, despite the criteria for carrying it out, induction is considered an invasive procedure that can have an impact on the experience of labor and birth, increasing waiting times and even obstetric complications, such as the risk of perineal laceration and postpartum hemorrhage ^(^ 25 ^)^ . 

 One of the “parturients” who had an elective cesarean section as her delivery route said that she had to remain isolated in the immediate postpartum period, making it a lonely time. The feeling that permeated this experience was similar to what can be called puerperal loneliness, characterized by the exacerbation of loneliness after childbirth, due to the impossibility of visits or the length of the hospital stay for her or the NB ^(^ 26 ^)^ . Even so, cesarean section proved to be a viable option for transsexual men who experienced pregnancy, and was the route of delivery chosen by four of the five “parturients”. 

The relationships that transsexual men establish with health professionals, especially those who accompany them during childbirth and the immediate postpartum period, have a significant influence on the care provided. There was a reproduction of delegitimization of the “parturient’s” gender identity, based on his identification with the female gender in the labeling of the NB in the delivery room, which generated a moment of intense stress, so that this situation can be characterized as transphobic violence, which demonstrates the lack of preparation in professional training and in the health institution.

 These situations can also occur when the newborn is registered at the health service. In Brazil, the Federal Supreme Court (FSC ordered the Ministry of Health, as of September 2021, to make changes to the Declaration of Live Birth (DLB) and the Live Birth Information System (SINASC) to include the category “parturient” in this document, regardless of the names of the parents. This makes it possible to collect data for the formulation of public policies and to respect the self-declared gender of ascendants ^(^ 27 ^)^ . 

 It has also been noted that after the increased use of in vitro fertilization techniques, adoptions, and other resources to guarantee parenthood among the LGBT population, especially transgender men, birth certificates began not to refer to the parents as “mother” and “father”, using the term “filiation” from 2017 with Provision No. 63 of the National Council of Justice (CNJ) ^(^ 28 ^-^ 29 ^)^ . In the environmental context, there was a report of the child being taken away from “the parturient” after cesarean delivery, as well as the occurrence of a situation of sexual violence when the user was under the effects of anesthesia. These situations are in addition to the various negative experiences that occur in the hospital environment, which are reflected in the suffering and mental illness of transsexual people. 

This environment, which is present at the time of childbirth and the immediate postpartum period, can be decisive as to what impressions a person will attribute to their experience. In this sense, sexual violence, suffered by one of the «parturients,» was present, in which he verbalized the occurrence of a “molestation” moments after the cesarean delivery by a health professional in an attempt to take advantage of the effects of spinal anesthesia to carry out the criminal action. In this situation, after reporting the violence he had suffered to the mother who accompanied him to the health service, “the parturient” suffered further violence when he was not listened to, and his experience was called into question as he was under the effects of medication.

The centrality of care was in the practice of using non-pharmacological techniques to cope with the pain of childbirth, while at the same time, there was disrespect for “the parturient” on the part of the health professional, making care fragile and violent. The use of non-pharmacological forms of pain relief during labor, such as music therapy, massage therapy, heat therapy, and the use of the Swiss ball, appeared to be strategies used by health professionals to provide support so that parturients could cope with the pain of labor since this is an event seen as one of the most significant experiences for the people involved.

“Health” expressed the “parturient’s” feelings about the health service provided during childbirth, noting dissatisfaction with the care provided by the institution, while recognizing the care taken by the health professional to offer a welcoming and supportive approach to dealing with the situations of violence present at the time. This generated a sense of hope in the client. We also noted the importance of the health professional’s role in making the parturient see him or her as a support in sharing their anguish, establishing a positive bond, and generating feelings of support and trust in coping with situations of violence. This fact highlights the need to consider the specificities of the parturient during obstetric care, based on recognizing and valuing their uniqueness, culture, and worldview.

 The “world view” is related to the breastfeeding process, revealing itself as a phenomenon that constitutes the subjects’ masculinity and can be carried out in the immediate postpartum period, with the aim of maintaining the child’s nutrition at this time of life. As for whether or not transsexual men «parturients» chose to breastfeed, the participants in this study were willing to do so in the immediate postpartum period, and sometimes the health team encouraged breastfeeding in the first hour of the NB’s life. In one of the “parturients”, who was in a heterosexual trans-centered stable relationship, the breastfeeding offered to the child took place through his partner, a transsexual woman, so that she could fulfill the social role of «mother», becoming responsible for this process until the fourth month of the NB’s life, and then the introduction of infant formula and the return to the use of testosterone by the transsexual man ^(^ 30 ^)^ . 

 The importance of the role of nurses in the process of health education on the risks and benefits of breastfeeding for parturient transsexual men who have not undergone the surgical procedure of masculinizing mastectomy is highlighted, throughout prenatal care, so that they can reflect and have the autonomy to decide whether or not to do it, understanding this event as part of their masculinity. To this end, spaces such as the rooming-in unit are ideal for nurses to provide this type of guidance ^(^ 31 ^)^ . 

 Obstetric-neonatal care, which is often marked by the violation of rights and cis-heteronormative culture, shows that as well as dealing with labor, which is already a challenging time, pregnant transsexual men also deal with prejudice and discrimination, which can be characterized as obstetric violence since they cause physical or psychological harm “to the parturient” ^(^ 32 ^)^ . 

 It should be noted that this study had limitations related to the number of participants, as it was a specific population and difficult to access, worsening during the COVID-19 pandemic, in a context of socioeconomic vulnerability. With a convenience sample, there were also some obstacles when it came to including transgender men in the study, as some of them didn’t get back in touch with the researcher or were not interested in taking part in a survey conducted by a cisgender person. In addition, the findings shed light on the recent debate in Brazil and around the world about transparency, as well as broadening the scope of men’s health care, taking into account different gender identities, as in the case of trans men, and the demands of sexual, reproductive and family health ^(^ 33 ^-^ 36 ^)^ . 

In order to minimize possible methodological limitations in the course of the research, the stages of approaching and selecting participants were strictly followed. The study was widely publicized to avoid bias in the recruitment strategy. The techniques used to understand the experiences of transsexual men during childbirth and postpartum were applied coherently, and the theoretical framework was used responsibly. In addition, the preliminary activities for the construction of the study were conducted in order to reinforce the theoretical sensitivity of the main researcher and the team. This included integrating the participants into the relevant contexts, investing in reading, participating in curricular subjects, and specific training in the area.

## Conclusion

This study showed that the social stigma that culturally emphasizes normal childbirth as dangerous, which generates pain and suffering, had a significant influence on the choice of delivery route among transsexual men, reinforcing that their experiences are affected by different situations of violence, whether moral, psychological, sexual, transphobic or due to negligence. The «parturient’s» worldview leads to the need for hospital care that recognizes their uniqueness. The environmental context can reveal greater vulnerability to situations that can have a negative impact on childbirth and postpartum care. Care is strengthened by strategies to support coping with situations intrinsic to labor, and health is weakened by violence and violations of rights.

## References

[B1] Pereira D. M. R., Araújo E. C., ATCSG Silva, Abreu P. D., Calazans J. C. C., Silva L. L. S. B. (2022). Scientific evidence on experiences of pregnant transsexual men. Texto Contexto Enferm.

[B2] Greenfield M., Darwin Z. (2021). Trans and non-binary pregnancy, traumatic birth, and perinatal mental health: a scoping review. Int J Transgend Health.

[B3] Malmquist A., Jonsson L., Wikström J., Nieminen K. (2019). Minority stress adds an additional layer to fear of childbirth in lesbian and bisexual women, and transgender people. Midwifery.

[B4] Ferreira M. J. S., Teixeira Z. M. (2020). Preliminary study of the portuguese version of the childbirth fear prior to pregnancy scale in a sample of university students. Rev Enferm Refer.

[B5] Almeida G. M. F., Nascimento T. F., Silva R. P. L., Bello M. P., Fontes C. M. B. (2021). Theoretical reflections of Leininger’s cross-cultural care in the context of Covid-19. Rev Gaucha Enferm.

[B6] Lenardt M. H., Michel T., Betiolli S. E., Seima M. D., Baran F. D. P., Brito C. S. (2021). Production of knowledge based on the Theory of Culture Care Diversity and Universality: documental research. Rev Bras Enferm.

[B7] Besse M., Lampe N. M., Mann E. S. (2020). Experiences with Achieving Pregnancy and Giving Birth Among Transgender Men: A Narrative Literature Review. Yale J Biol Med [Internet].

[B8] Rocon P. C., Wandekoken K. D., Barros M. E. B., Duarte M. J. O., Sodré F. (2019). Access to health by the transsexual population in brazil: between the lines of the integrative review. Trab Educ Saúde.

[B9] Patias N. D., Hohendorff J. (2019). Quality criteria for qualitative research articles. Psicol Estud.

[B10] Minayo M. C. S. (2012). Qualitative analysis: theory, steps and reliability. Cienc Saude Coletiva.

[B11] Nascimento L. C., Silva T. C., DPODV Tafner, Oliveira V. J., Viegas S. M. F. (2023). The pandemic changes daily life and ways of living: technosociality and user/families experiences. Rev Bras Enferm.

[B12] Fontanella B. J. B., Luchesi B. M., Saidel M. G. B., Ricas J., Turato E. R., Melo D. G. (2011). Sampling in qualitative research: a proposal for procedures to detect theoretical saturation. Cad Saude Publica.

[B13] Minayo M. C. S. (2017). Sampling and saturation in qualitative research: consensuses and controversies. Rev Pesqui Qualitat [Internet].

[B14] Einhardt K. G., Bordignon S. S., Tomaschewski-Barlem J. G., Castanheira J. S., Rocha L. P., Carvalho D. P. (2022). Nursing students: the use of digital social network to profile nurses. Rev Bras Enferm.

[B15] Reinert M. (1990). A Methodology of Textual Data Analysis and an Application: Aurélia by Gérard de Nerval. Bull Sociol Methodol.

[B16] Soares S. S. S., Costa C. C. P., Carvalho E. C., Queiroz A. B. A., Peres P. L. P., NVDDO Souza (2022). Teaching Iramuteq for use in qualitative research according to YouTube videos: an exploratory-descriptive study. Rev Esc Enferm USP.

[B17] Sousa Y. S. O. (2021). The Use of the Iramuteq Software: Fundamentals of Lexicometry for Qualitative Research. Estud Pesqui Psicol.

[B18] Camargo B. V., Justo A. M. (2018). Tutorial para uso do software Interface de R pour les Analyses Multidimensionnelles de Textes et de Questionnaires [Internet].

[B19] Sousa Y. S. O., Gondim S. M. G., Carias I. A., Batista J. S., Machado K. C. M. (2020). The use of the Iramuteq software in the interview data analysis. Pesqui Prat Psicossoc [Internet].

[B20] Melo B. L. P. L., Moreira F. T. L. S., Alencar R. M., Magalhães B. C., Cavalcante E. G. R., Maia E. R. (2022). Obstetric violence in the light of the theory of culture care diversity and universality. Rev Cuidarte.

[B21] Brasil (2013). Resolução nº 466, de 12 de dezembro de 2012. Dispõe sobre diretrizes e normas regulamentadoras de pesquisas envolvendo seres humanos.

[B22] Farias C. M. L., Giovanella L., Oliveira A. E., Santos  E. T. (2019). Waiting time and absenteeism in the secondary care: a challenge for universal health systems. Saude Debate.

[B23] Travancas L. J., Vargens O. M. C. (2020). Factors that generate fear of childbirth: An integrative review. Rev Enferm UFSM.

[B24] Obedin-Maliver J., Makadon H. J. (2015). Transgender men and pregnancy. Obstet Med.

[B25] Koch D. M., Rattmann Y. D. (2021). Misoprostol for labor induction: pharmacoepidemiological approach and evaluation of the impact on cesareans delivery reduction. Rev Bras Cien Saude.

[B26] Paixão G. P. D. N., Campos L. M., Carneiro J. B., Fraga C. D. S. (2021). Maternal solitude before the new guidelines in SARS-COV-2 times: a Brazilian cutting. Rev Gaucha Enferm.

[B27] Ministério da Saúde (BR) (2021). Secretaria de Vigilância em Saúde. Nota técnica n. 195/2021-CGIAE/DASNT/SVS/MS [Internet].

[B28] Severiano J. C. (2021). O reconhecimento extrajudicial da parentalidade socioafetiva conforme as modificações do provimento no 63/2017 do CNJ.

[B29] Barros R. S., Mendonça E. G. (2021). Anais do 21° Simpósio de TCC do Centro Universitário ICESP; 2021; Águas Claras.

[B30] Carvalho M. P., Santos L. M. T., Abilio C.  (2021). O Aleitamento Materno. Rev Cient Multidiscip Núcleo Conhec.

[B31] Raminelli M., Hahn SR (2019). Medications in breastfeeding: what evidence is there?. Cienc Saude Colet.

[B32] Barrera D. C., Moretti-Pires R. O. (2021). From Obstetric Violence to Empowerment of Pregnant People in the Doulas’s Work. Rev Estud Feministas.

[B33] Pfeil C. L., Pfeil B. L. (2023). Em defesa de parentalidades transmasculinas. Rev Bras Estud Homocultura.

[B34] Silva F. A., Gravidez Latini C. (2023). Aborto e Parentalidade nas Transmasculinidades. Rev Bras Estud Homocultura.

[B35] Sousa A. R. (2020). Produce health care for men and their masculinities: a priority. REVISA.

[B36] Sousa A. R., Araújo I. F. M., Borges C. C. L., Oliveira J. A., Almeida M. S., Caribé W. (2021). Men’s health in the covid-19 pandemic: brazilian panorama. Rev Baiana Enferm.

