# Supplementary material for: Transsexual men’s experiences of childbirth and postpartum in the light of transcultural care
Source: Rev Lat Am Enfermagem. 2024 Nov 22;32:e4212. doi: 10.1590/1518-8345.7040.4212 (PMC11654047; doi:10.1590/1518-8345.7040.4212)
Supplement: Translation

# Translation: Experiencias de hombres transexuales en el parto y posparto a la luz del cuidado transcultural*

**DOI:** 10.1590/1518-8345.7040.4211

**Published:** 2024-11-15

**Authors:** Danilo Martins Roque Pereira, Ednaldo Cavalcante de Araújo, Sheyla Costa de Oliveira, Anderson Reis de Sousa, Mariana Mercês Mesquita Espíndola, Dante Eduardo Barbosa de Lemos

**Affiliations:** ^1^ Universidade Federal de Pernambuco, Recife, PE, Brasil.; ^2^ Becario de la Coordenação de Aperfeiçoamento de Pessoal de Nível Superior (CAPES), Brasil.; ^3^ Universidade Federal da Bahia, Salvador, BA, Brasil.; ^4^ Universidade Federal de Pernambuco, Departamento de Enfermagem, Recife, PE, Brasil.

**Keywords:** Personas Transgénero, Parto, Salud del Hombre, Atención Primaria de Salud, Minorías Sexuales y de Género, Enfermería

## Abstract

revelar las experiencias de hombres transexuales durante el parto y posparto a la luz de la Teoría de la Diversidad y Universalidad del Cuidado Cultural.

estudio cualitativo y descriptivo operacionalizado por el método de estudio de casos múltiples. Recolección de datos realizada por medio de un muestreo intencional compuesto por cinco hombres transexuales, seleccionados con base en criterios de conveniencia y disponibilidad. Las entrevistas fueron transcritas íntegramente y los resultados fueron organizados y adaptados al Modelo *Sunrise* .

la mayoría de los participantes son “primíparos” y con parto por cesárea. Al adaptar el Modelo *Sunrise* , se observó el estímulo a la medicalización y el manejo mecanicista del parto; miedo al parto natural; violencia perpetrada contra hombres transexuales producto de la dificultad de acceso a la información por parte del hombre embarazado y de la atención obstétrica no calificada a las necesidades del público, resultando en un cuidado debilitado, con insatisfacción con el servicio de salud.

las experiencias de los hombres transexuales durante el parto y posparto involucran una mezcla de vivencias que generan daño, especialmente cuando se vinculan a situaciones de violencia transfóbica y vulneración de derechos.

## Destacados

**(1)** Promueve la reflexión sobre la atención obstétrica a hombres transexuales.

**(2)** Influencia del estigma social sobre el parto natural en la elección de la vía de parto.

**(3)** Necesidad de una atención hospitalaria que reconozca la singularidad del parturiento.

**(4)** El contexto ambiental puede revelar mayores situaciones negativas en la asistencia.

**(5)** El cuidado se potencia con estrategias de afrontamiento de las situaciones del parto.

## Introducción

 Desde el punto de vista de la salud integral, el proceso de embarazo en hombres transexuales puede resultar en experiencias positivas y negativas, ya que, en su mayoría, algunos de ellos han conservado los órganos del sistema reproductor, como la vagina, el útero, las trompas y los ovarios, con capacidad de gestar y dar a luz, haciendo imprescindible la atención de salud ginecológica y obstétrica, como garantía de los derechos sexuales y reproductivos, la seguridad y la protección embarazo-puerperio ^(^ 1 ^)^ . 

 Se sabe que, en Australia, entre 2013 y 2018, hubo un total de 205 hombres trans que dieron a luz. A su vez, en Brasil, las personas trans y no binarias que quedan embarazadas no son identificadas en los datos de los servicios de salud, debido a la imposibilidad de completar la pregunta de “identidad de género” en lugares de referencia para el cuidado obstétrico, como los centros de parto o maternidades. Esto impide analizar las diferentes experiencias durante el parto y el puerperio, así como visibilizar las desigualdades en el acceso y el impacto en la salud integral individual ^(^ 2 ^)^ . 

Faltan estudios nacionales e internacionales que aborden el tema aquí discutido, en particular, existe un vacío importante en el ámbito de la Enfermería en cuanto a la atención a la salud de hombres transexuales durante el ciclo embarazo-puerperio, ya que ese cuidado se discute durante la formación profesional desde una perspectiva cisheteronormativa, contribuyendo a una visión limitada del cuidado y excluyendo experiencias y necesidades específicas de este grupo social. De esta manera, la realización de esta investigación podría apoyar una práctica avanzada en Enfermería, con el fin de considerar las particularidades socioculturales de este segmento poblacional durante el parto y el posparto inmediato.

 Se destaca que, comúnmente, los hombres transexuales, durante el embarazo, expresan a los profesionales de la salud malestar con su propio cuerpo y/o sus genitales, y que existe una sensación de malestar con situaciones cotidianas en el cuidado de la salud, como los exámenes ginecológicos y el examen del tacto vaginal a partir de la semana 36 de embarazo. Además, suponen que estos exámenes pueden realizarse sin su consentimiento, incluso durante el parto, lo que contribuye a aumentar el miedo y la ansiedad y a no buscar servicios de salud ^(^ 3 ^-^ 4 ^)^ . En este sentido, cabe señalar que dichos exámenes contribuyen a la identificación de problemas de salud, e incluso a evitar un posible parto prematuro, ya que también tienen como objetivo analizar la pelvis, con el fin de evaluar la estructura de la vagina y del cuello uterino. 

 En este contexto, la Teoría de la Diversidad y Universalidad del Cuidado Cultural (TDUCC) de Madeleine Leininger, en su modelo conceptual *Sunrise* , propuso considerar el cuidado del ser humano de manera congruente con el conocimiento de su cultura y de su visión del mundo, descubriendo el significado del cuidado transcultural, basado en prácticas específicamente dirigidas a cada cultura y sus factores de influencia en la prestación de atención de salud. La teórica interpreta la existencia de una diversidad y universalidad cultural con un enfoque en estructurar el cuidado, para que la persona pueda ser atendida de manera satisfactoria y humanística, y los conceptos de “Cultura”, “Visión del mundo”, “Contexto ambiental”, “Cuidado” y “Salud” son fundamentales para entender este estudio ^(^ 5 ^-^ 6 ^)^ . 

 Se destaca la importancia de reducir las desigualdades y disparidades en salud, promover la equidad en el cuidado y garantizar los derechos humanos y la justicia social para alcanzar los Objetivos de Desarrollo Sostenible ^(^ 7 ^-^ 8 ^)^ . En este sentido, este estudio posibilita una reflexión sobre la atención obstétrica a hombres transexuales durante el parto y posparto que promueva cambios significativos en los procesos de trabajo en diferentes escenarios de práctica profesional pública o privada. Ante lo anterior, se formuló la siguiente pregunta orientadora: ¿cuáles son las experiencias de los hombres transexuales durante el parto y el posparto? Para ello, el objetivo fue revelar las vivencias de hombres transexuales durante el parto y posparto a la luz de la Teoría de la Diversidad y Universalidad del Cuidado Cultural. 

## Método

### Diseño y lugar del estudio

 Se trata de un estudio cualitativo ^(^ 9 ^-^ 10 ^)^ y operacionalizado por el método de Estudio de Casos Múltiples ^(^ 11 ^)^ . El escenario investigado está vinculado a la *Secretaria Estadual de Saúde* , por medio de la *Coordenação Estadual de Atenção Integral à Saúde de Lésbicas, Gays, Bissexuais, Travestis e Transexuais* (LGBT) de Pernambuco (PE), y a nivel nacional con personas vinculadas a la *Aliança Nacional LGBT* . 

### Definición de la muestra

 En el estudio participaron cinco hombres transexuales “parturientos”. El muestreo fue intencional ^(^ 12 ^)^ , compuesto por participantes seleccionados con base en criterios de conveniencia y disponibilidad, luego de establecer los siguientes criterios de inclusión: ser hombre transexual con experiencia en parto vaginal o cesárea, y tener 18 años de edad o más. Los criterios de exclusión fueron: hombres transexuales que vivenciaron el trabajo de parto y que no contaban con recursos tecnológicos como computadora, celular, *Internet* , entre otros, para posibilitar la realización de la entrevista. La inclusión de los participantes se hizo mediante inserción progresiva, basada en la saturación teórica de las respuestas a entrevistas semiestructuradas ^(^ 13 ^)^ . 

### Recolección de datos

 La recolección de datos se realizó en los meses de septiembre y octubre de 2021. Para ello, se utilizó la técnica de entrevistas individuales ^(^ 13 ^)^ , en modalidad virtual (remota), a través de la plataforma *Google Meet* ^®^ , en un solo encuentro, con un guion semiestructurado, compuesto por preguntas cerradas relacionadas con características sociodemográficas y por preguntas abiertas relacionadas con el fenómeno empírico investigado, a saber: 1. Cuénteme su experiencia durante el parto y posparto; 2. Cuénteme sobre la atención médica brindada por el equipo de salud durante su parto y posparto; 3. Si eligió amamantar posparto, cuénteme cómo fue esta experiencia; 4. ¿Conoce otros hombres transexuales que hayan quedado embarazados o estén embarazados? Cuénteme sobre las experiencias que conoció. La caracterización sociodemográfica de los participantes se realizó tras proporcionar un cuestionario antes de las entrevistas, durante su reclutamiento a través del contacto con las instituciones asociadas, por medio de las principales redes sociales digitales de Brasil: *Facebook* ^®^ , *Instagram* ^®^ y *WhatsApp* ® ^(^ 2022 ^)^ . 

 Las anuencias para el Término de Consentimiento Libre e Informado (TCLE, por sus siglas en portugués) y para el Término de Autorización de Uso de Imagen y Testimonio estaban disponibles para firma en la plataforma *Google Forms* ^®^ ( *on-line* ). Para cada entrevista se solicitó que se encendiera la cámara del celular o computadora para capturar imágenes, lenguaje corporal y entonaciones de voz utilizando la plataforma *Google Meet* ^®^ , con un tiempo promedio de una hora para cada entrevista. 

 Las entrevistas fueron realizadas por el investigador principal, quien es miembro de la comunidad LGBT y tiene experiencia en la realización de estudios cualitativos, contribuyendo al proceso de recolección de la información, que luego fue transcrita en su totalidad y puesta a disposición de los participantes para la validación del contenido y realización de ajustes y correcciones, si fuera necesario. En esta etapa, todos los participantes leyeron el material y no sugirieron cambios en el contenido. Todo el material fue sometido a análisis lexicográfico clásico utilizando el Método Reinert ^(^ 15 ^)^ de clasificación de segmentos de texto instrumentado por el *software* gratuito *Interface de R pour les Analyses Multidimensionnelles de Textes et de Questionnaires* (IRAMuTeQ) versión 7.0 ^(^ 16 ^)^ . Utilizando el IRAMuTeQ, fue posible categorizar los datos evaluando la similitud ^(^ 17 ^-^ 19 ^)^ . 

### Procesamiento y análisis de los datos

 Los hallazgos centrales encontrados en el Estudio de Casos Múltiples realizado fueron discutidos teóricamente con base en los supuestos de la TDUCC ^(^ 20 ^)^ . El análisis de los datos tuvo como base la TDUCC, propuesta por Madeleine Leininger, que aborda la necesidad de que los profesionales consideren el contexto cultural en el que el individuo está inserto, brindando cuidados armoniosos a aquella identidad local, y enumerando los cinco conceptos que la sostienen: cultura, visión del mundo, contexto ambiental, cuidado y salud; asociado con la literatura científica relacionada al tema ^(^ 20 ^)^ . 

### Aspectos éticos

 Este estudio fue sometido a la aprobación del Comité de Ética en Investigación de la *Universidade Federal de Pernambuco* (UFPE), bajo CAAE nº 47777421.0.0000.5208 y dictamen nº 4.862.503/2021, siguiendo las recomendaciones de la Resolución nº 466 de 12 de diciembre de 2012 del Consejo Nacional de Salud de Brasil ^(^ 21 ^)^ . 

## Resultados

 La Figura 1 muestra la disposición de los casos múltiples de los hombres transexuales “parturientos” del estudio, identificados por seudónimos autodeclarados en el momento de las entrevistas, según el perfil embarazo-parto. 

**Figura 1.** Casos múltiples de los hombres transexuales participantes en el estudio (n = 5). Recife, PE, Brasil, 2023

 La Figura 2 presenta la agrupación de los registros más significativos por concepto listados a partir de la adaptación del Modelo *Sunrise* , propuesto por la Teoría de la Diversidad y Universalidad del Cuidado Cultural (TDUCC), ubicando los discursos de acuerdo con las experiencias de los hombres transexuales durante el parto y posparto. Así, los relatos exponen el miedo al parto, la violencia obstétrica, transfóbica, moral, psicológica, sexual o por negligencia, entre otros, como se presenta a continuación. 

**Figura 2.** Experiencias atribuidas al parto y posparto inmediato por hombres transexuales adaptadas al Modelo *Sunrise* . Recife, PE, Brasil, 2023

 La Figura 3 muestra el resultado de la construcción de la adaptación del Modelo *Sunrise* , traducido en ideas claves y más recurrentes en los discursos de los hombres transexuales parturientos, destacando los aspectos intrínsecos del parto y posparto vividos por estos sujetos, para ser considerados para el reconocimiento del cuidado transcultural. La Figura 4 muestra una relación entre los factores culturales y los elementos presentes en los discursos de los individuos que participaron en el estudio. 

**Figura 3.** Adaptación del Modelo *Sunrise* . Valores que influyen en el cuidado de la salud de los parturientos. Recife, PE, Brasil, 2023

**Figura 4.** Factores culturales que influyen en los cuidados de salud y los elementos presentes en el discurso de los parturientos. Recife, PE, Brasil, 2023

## Discusión

Se destaca la ausencia de literatura científica nacional e internacional que aborde las especificidades de los hombres transexuales durante el embarazo, el parto y el puerperio, reafirmando la necesidad de este estudio, que engloba a la Obstetricia y la Enfermería en las discusiones sobre las relaciones entre salud, género y sexualidades. Teniendo en cuenta lo anterior, este estudio presenta aportes e implicaciones relevantes en estas áreas y en el avance del conocimiento científico en la atención prenatal y obstétrica a las diferentes identidades de género, especialmente en lo que respecta a la visibilización de las experiencias de los hombres transexuales durante el parto y el posparto inmediato, bajo el análisis de los conceptos de “Cultura”, “Visión del mundo”, “Contexto ambiental”, “Cuidado” y “Salud” propuestos por la TDUCC de Madeleine Leininger.

 En el ítem referido a “cultura”, se registró la percepción que tenían los hombres transexuales respecto al parto natural y los hechos de violencia ocurridos en ese momento, en ocasiones naturalizados. En este sentido, el estigma social que culturalmente considera peligroso el parto natural, que genera dolor y sufrimiento, influye significativamente en las experiencias de los hombres transexuales. Este aspecto corrobora lo planteado por Leininger, es decir, que la cultura puede guiar al individuo o a un grupo en sus acciones y construir la forma como le gustaría ser cuidado. Parte de este sentimiento está relacionado con la atención de salud ofrecida por el *Sistema Único de Saúde* (SUS), muchas veces vista como no calificada, ya que se cree que la atención ocurre después de largos períodos de espera, lo que justifica la elección de la cesárea, incluso frente a condiciones para realizar un parto natural, considerando la vía quirúrgica como rápida y segura ^(^ 22 ^)^ . 

 Es de destacar que, con la medicalización del parto, se generalizó socialmente que los efectos de los medicamentos y de los procedimientos técnicos y tecnológicos realizados por los profesionales de la salud son más eficientes que la propia fisiología del cuerpo. La evidencia científica reporta que los principales factores que desencadenan el miedo al parto natural entre los hombres transexuales son los relatos de dolor de familiares y amigos que han pasado por este momento, la ansiedad relacionada con el sufrimiento durante el parto natural, la inseguridad de no poder completar el parto y el temor de que cause complicaciones al recién nacido (RN) ^(^ 23 ^)^ . El miedo a este momento también puede estar relacionado con cambios en el canal vaginal (la pared de la vagina está formada por tres capas: mucosa, muscular y adventicia), por el uso de testosterona durante el proceso de afirmación de género y preocupaciones por cuidados insuficientes, como la falta de recursos en los servicios de salud, o relacionados con la competencia transcultural de los profesionales en la asistencia al parto de un hombre transexual, dada la incipiente discusión sobre el tema en los cursos de formación en salud ^(^ 3 ^-^ 4 ^,^ 24 ^)^ . 

 Una de las experiencias de uno de los participantes en el estudio, que optó por un parto natural, fue el procedimiento de inducción del parto y episiotomía. Se observó la falta de información por parte del usuario sobre de qué se trataba, y la toma de decisiones en la realización de los procedimientos sin el consentimiento explícito e informado del “parturiento” también puede caracterizarse como violencia obstétrica. Es de destacar que, a pesar de los criterios para realizarla, la inducción es considerada un procedimiento invasivo, y puede impactar la experiencia del parto y del nacimiento, aumentando el tiempo de espera, e incluso derivando en complicaciones obstétricas, como el riesgo de laceración perineal y hemorragia posparto ^(^ 25 ^)^ . 

 Uno de los “parturientos”, a quien se le realizó una cesárea electiva, afirmó que tuvo que permanecer aislado en el posparto inmediato, por lo que vivió un momento de soledad. El sentimiento que impregnó esta experiencia fue similar a lo que se puede llamar “soledad puerperal”, caracterizada por la exacerbación de la soledad después del parto, debido a la imposibilidad de visitas o a la duración de su internación o de la del recién nacido ^(^ 26 ^)^ . Aun así, la cesárea demostró ser una opción viable para los hombres transexuales que experimentaron un embarazo, siendo la vía de parto elegida por cuatro de los cinco “parturientos”. 

Las relaciones que los hombres transexuales establecen con los profesionales de la salud, especialmente con aquellos que los acompañan durante el parto y el posparto inmediato, han influido significativamente en el cuidado brindado. Se reproduce una deslegitimación de la identidad de género del “parturiento”, a partir de su identificación por el género femenino en la etiqueta del recién nacido en la sala de parto, algo que generó un momento de intenso estrés, caracterizándose esta situación como violencia transfóbica, lo que demuestra la falta de preparación en la formación profesional y de la institución de salud.

 Estas situaciones pueden ocurrir, aún, en el momento del registro del recién nacido en el servicio de salud. Así, en Brasil, el Supremo Tribunal Federal (STF) determinó que el *Ministério da Saúde* , a partir del mes de septiembre de 2021, realizara modificaciones en la Declaración de Nacido Vivo (DNV) y en el Sistema de Información de Nacidos Vivos (SINASC), para que en este documento figure la categoría “parturiente”, independientemente del nombre de los genitores. Esto permite recolectar datos para la formulación de políticas públicas y el respeto a la autodeclaración de género de los ascendientes ^(^ 27 ^)^ . 

 También se puede observar que, tras el incremento en el uso de técnicas de fertilización *in vitro* , adopciones y otros recursos para garantizar la parentalidad entre la población LGBT, especialmente entre los hombres transexuales, el certificado de nacimiento ya no hace referencia a los genitores como “madre” y “padre”, utilizando el término “filiación” desde 2017, con la Disposición n° 63 del *Conselho Nacional de Justiça* (CNJ) ^(^ 28 ^-^ 29 ^)^ . El contexto ambiental presentó el relato de separación del bebé del “parturiento” luego de un parto por cesárea, así como la ocurrencia de una situación de violencia sexual cuando el usuario se encontraba bajo los efectos de la anestesia. Estas situaciones se suman a las diversas experiencias negativas que suceden en el ambiente hospitalario, con consecuencias en el sufrimiento y desencadenantes de enfermedades mentales en las personas transgénero. 

Este ambiente que está presente en el momento del parto e inmediatamente en el posparto puede ser decisivo en qué impresiones atribuirá la persona a su experiencia. En ese sentido, se hizo presente la violencia sexual que sufrió uno de los “parturientos”, quien verbalizó la ocurrencia de un abuso sexual momentos después del parto por cesárea por parte de un profesional de la salud, en un intento de aprovechar los efectos de la anestesia para cometer una acción criminal. En esta situación, luego de denunciar la violencia sufrida a la genitora que lo acompañaba en el servicio de salud, el “parturiento” sufrió más violencia aún al no ser escuchado, poniendo en duda su experiencia, ya que se encontraba bajo los efectos de una medicación.

La centralidad del cuidado estuvo en la práctica de utilizar técnicas no farmacológicas para afrontar los dolores del parto, al mismo tiempo que hubo irrespeto al “parturiento” por parte del profesional de la salud, tornando la asistencia frágil y violenta. El uso de formas no farmacológicas de alivio del dolor durante el trabajo de parto, como la musicoterapia, la masoterapia, la termoterapia y el uso de la pelota suiza, surgieron como estrategias utilizadas por los profesionales de la salud con el fin de brindar apoyo para las personas parturientas, ya que se trata de un evento considerado como una de las experiencias más significativas para quienes participan.

“Salud” expresó el sentimiento del “parturiento” acerca del servicio de salud brindado durante el parto, observándose la insatisfacción con la asistencia brindada por la institución, al mismo tiempo que reconoce el cuidado del profesional de salud al ofrecer acogida y apoyo para hacer frente a las situaciones de violencia presentes en aquel momento. Esto generó un sentimiento de esperanza en el paciente. También se observó la importancia del papel del profesional de la salud para que el “parturiento” lo viera como un soporte para compartir sus angustias, estableciendo un vínculo positivo y generando sentimientos de apoyo y confianza ante situaciones de violencia. Este hecho resalta la necesidad de considerar las especificidades de la persona parturienta durante la atención obstétrica, a partir del reconocimiento y de la valoración de su singularidad, su cultura y su visión del mundo.

 La “visión del mundo” está relacionada con el proceso de lactancia, revelándose como un fenómeno que constituye la masculinidad de los sujetos y posible de ser realizada en el posparto inmediato, con el objetivo de mantener la nutrición del niño en ese momento de la vida. Respecto a si los hombres transexuales “parturientos” eligieron o no amamantar, se verifica que los participantes en este estudio estaban dispuestos a hacerlo en el posparto inmediato, y en ocasiones la lactancia fue incentivada por el equipo de salud en la primera hora de vida del RN. En el caso de uno de los “parturientos”, quien se encontraba en una relación estable transcéntrica heterosexual, la lactancia ofrecida al bebé se produjo por medio de su pareja, una mujer transexual, para que ella cumpliera el rol social de “madre”, convirtiéndose en la responsable de este proceso hasta el cuarto mes de vida del RN, para luego comenzar la introducción de la fórmula láctea infantil y el retorno del uso de testosterona por parte del hombre transexual ^(^ 30 ^)^ . 

 Se destaca la importancia del papel del enfermero en el proceso de educación en salud respecto a la orientación sobre los riesgos y beneficios de la lactancia para hombres transexuales parturientos que no han sido sometidos al procedimiento quirúrgico de mastectomía masculinizante durante todo el control prenatal, para que puedan reflexionar y tener autonomía para decidir si realizarla o no, entendiendo este evento como constitutivo de su masculinidad. Para ello, espacios como el alojamiento conjunto se vuelven aptos para que los enfermeros realicen este tipo de orientación ^(^ 31 ^)^ . 

 La atención obstétrico-neonatal, a menudo marcada por la violación de derechos y por la cultura cisheteronormativa, muestra que, además de lidiar con el parto, un momento que ya es desafiante, los hombres transexuales embarazados también enfrentan prejuicios y discriminación, que pueden caracterizarse como violencia obstétrica, ya que causan daño físico o psicológico al “parturiento” ^(^ 32 ^)^ . 

 Cabe señalar que este estudio tuvo limitaciones relacionadas con el número de participantes, al tratarse de una población específica de difícil acceso, lo que se agravó durante el período de la pandemia de COVID-19, en un contexto de vulnerabilidad socioeconómica. Ante una muestra por conveniencia, también hubo algunos obstáculos en la inclusión de hombres transexuales en el estudio, ya que algunos de ellos no retomaron el contacto con el investigador o no estaban interesados en participar en la investigación realizada por una persona cisgénero. Además, los hallazgos arrojan luz sobre el reciente debate en Brasil y en el mundo sobre las transparentalidades, así como amplían el alcance de la atención de salud de los hombres, considerando las diferentes identidades de género, como en el caso de los hombres trans y las demandas de salud sexual, reproductiva y familiar ^(^ 33 ^-^ 36 ^)^ . 

Para minimizar posibles limitaciones metodológicas durante la investigación, se siguieron estrictamente los pasos de abordaje y selección de los participantes. Se realizó una amplia difusión del estudio para evitar sesgos en la estrategia de reclutamiento. Las técnicas utilizadas para comprender las experiencias de los hombres transexuales durante el parto y posparto se aplicaron de manera coherente, y el marco teórico se utilizó de manera responsable. Además, se realizaron actividades preliminares para la construcción del estudio, con el objetivo de reforzar la sensibilidad teórica del investigador principal y del equipo. Esto incluyó la integración de los participantes en contextos relevantes, inversión en lectura, participación en materias curriculares y capacitaciones específicas en el área.

## Conclusión

Este estudio demostró que el estigma social que culturalmente enfatiza el parto normal como peligroso, que genera dolor y sufrimiento, ejerció una influencia significativa en la elección de la vía de parto entre los hombres transexuales, reforzando que las experiencias vividas se ven afectadas por diferentes situaciones de violencia, ya sea moral, psicológica, sexual, transfóbica o por negligencia. La visión del mundo del “parturiento” conduce a la necesidad de una atención hospitalaria que reconozca su singularidad. El contexto ambiental, por otro lado, puede revelar una mayor vulnerabilidad a la ocurrencia de situaciones que podrían generar impactos negativos en la atención del parto y posparto. El cuidado se potencia mediante la realización de estrategias que apoyen el enfrentamiento de situaciones intrínsecas al trabajo de parto y a la salud, sin embargo, por medio de la violencia y de la vulneración de derechos, se debilita.

# Translation: Experiências de homens transexuais no parto e pós-parto à luz do cuidado transcultural*

**DOI:** 10.1590/1518-8345.7040.4213

**Published:** 2024-11-15

**Authors:** Danilo Martins Roque Pereira, Ednaldo Cavalcante de Araújo, Sheyla Costa de Oliveira, Anderson Reis de Sousa, Mariana Mercês Mesquita Espíndola, Dante Eduardo Barbosa de Lemos

**Affiliations:** ^1^ Universidade Federal de Pernambuco, Recife, PE, Brasil.; ^2^ Bolsista da Coordenação de Aperfeiçoamento de Pessoal de Nível Superior (CAPES), Brasil.; ^3^ Universidade Federal da Bahia, Salvador, BA, Brasil.; ^4^ Universidade Federal de Pernambuco, Departamento de Enfermagem, Recife, PE, Brasil.

**Keywords:** Pessoas Transgênero, Parto, Saúde do Homem, Atenção Primária à Saúde, Minorias Sexuais e de Gênero, Enfermagem

## Abstract

desvelar as experiências de homens transexuais durante o parto e pós-parto à luz da Teoria da Diversidade e Universalidade do Cuidado Cultural.

estudo qualitativo e descritivo operacionalizado pelo método de estudo de casos múltiplos. Coleta de dados realizada por meio da amostragem do tipo intencional composta por cinco homens transexuais, selecionados por critérios de conveniência e disponibilidade. As entrevistas foram transcritas na íntegra e os resultados foram organizados e adaptados ao Modelo *Sunrise* .

a maioria dos participantes são “primíparos”, e com parto cesáreo. Na adaptação do Modelo *Sunrise* , observou-se o estímulo à medicalização e manejo mecanicista do parto; medo do parto natural; violência perpetrada contra os homens transexuais resultantes da dificuldade de acesso à informação por parte do homem gestante e atenção obstétrica não qualificada às necessidades do público, repercutindo em cuidado fragilizado, com insatisfação frente ao serviço de saúde.

as experiências de homens transexuais durante o parto e pós-parto perpassam por um misto de vivências, geradoras de danos, sobretudo, quando vinculadas às situações de violência transfóbica e violação de direitos.

## Destaques

**(1)** Possibilita reflexão sobre a assistência obstétrica aos homens transexuais.

**(2)** Influência do estigma social sobre o parto normal na escolha da via de parto.

**(3)** Necessidade de atenção hospitalar que reconheça a singularidade do parturiente.

**(4)** O contexto ambiental pode revelar maiores situações negativas na assistência.

**(5)** O cuidado é potencializado por estratégias de enfrentamento às situações do parto.

## Introdução

 Do ponto de vista da saúde integral, o processo de gestação nos homens transexuais pode resultar em experiências positivas e negativas, visto que, em sua maioria, alguns deles têm preservado os órgãos do sistema reprodutor, a exemplo da vagina, útero, tubas uterinas e ovários, com a capacidade de gestar e parir, tornando-lhes imprescindíveis a atenção à saúde ginecológica e obstétrica, como garantia de direitos sexuais e reprodutivos, a segurança e a proteção gravídico-puerperal ^(^ 1 ^)^ . 

 Sabe-se que na Austrália, entre os anos de 2013 e 2018, registrou-se um total de 205 homens trans que pariram; no Brasil, por sua vez, pessoas trans e não-binárias que engravidam não são identificadas nos dados de serviços de saúde pela impossibilidade de preenchimento do quesito “identidade de gênero” em locais de referência para o cuidado obstétrico, a exemplo dos centros de parto ou maternidades. Isso impossibilita analisar as diferentes experiências durante o parto e o puerpério, bem como tornar visíveis as desigualdades no acesso e os reflexos na saúde integral individual ^(^ 2 ^)^ . 

Verifica-se a incipiência de estudos nacionais e internacionais abordando a temática aqui discutida. Em particular, há uma lacuna significativa no âmbito da Enfermagem, em relação à assistência à saúde de homens transexuais durante o ciclo gravídico-puerperal, uma vez que esse cuidado é discutido durante a formação profissional a partir de uma perspectiva cis-heteronormativa, contribuindo com uma visão limitada do cuidado, excluindo experiências e necessidades específicas deste grupo social. Dessa forma, a realização desta pesquisa poderá subsidiar uma prática avançada em Enfermagem de forma a considerar as particularidades socioculturais deste segmento populacional durante o parto e pós-parto imediato.

 Destaca-se que, corriqueiramente, os homens transexuais, no período gravídico, manifestam aos profissionais da saúde desconforto com seu próprio corpo e/ou com a sua genitália, e que há a sensação de incômodo com situações cotidianas no cuidado à saúde, como os exames ginecológicos e o de toque vaginal a partir 36ª semana de gestação. Além disso, presumem que esses exames podem ser realizados sem o seu consentimento, inclusive, no trabalho de parto, o que contribui para o aumento do medo e da ansiedade e da não busca pelos serviços de saúde ^(^ 3 ^-^ 4 ^)^ . A esse respeito, ressalta-se que tais exames contribuem na identificação de problemas de saúde, e até para evitar um possível parto prematuro, pois objetivam analisar também a pélvis, de modo a avaliar a estrutura da vagina e do colo do útero. 

 Nesse contexto, a Teoria da Diversidade e Universalidade do Cuidado Cultural (TDUCC) de Madeleine Leininger, em seu modelo conceitual, o *Sunrise* , propôs considerar o cuidado ao ser humano de modo congruente com o conhecimento de sua cultura e visão de mundo, descobrindo o significado de um cuidado transcultural, a partir de práticas voltadas especificamente para cada cultura e seus fatores influenciadores na prestação da assistência à saúde. A teorista interpreta a existência de uma diversidade e universalidade cultural com foco na estruturação do cuidado para que a pessoa possa ser assistida de modo satisfatório e humanístico, sendo os conceitos de “Cultura”, “Visão de mundo”, “Contexto ambiental”, “Cuidado” e “Saúde” fundamentais para a compreensão deste estudo ^(^ 5 ^-^ 6 ^)^ . 

 Ressalta-se a importância de diminuir as desigualdades e as disparidades em saúde, promover a equidade no cuidado e possibilitar a garantia dos direitos humanos e da justiça social para se alcancem os Objetivos do Desenvolvimento Sustentável ^(^ 7 ^-^ 8 ^)^ . Nesse sentido, este estudo possibilita a reflexão sobre a assistência obstétrica aos homens transexuais no parto e pós-parto que promova as mudanças significativas dos processos de trabalho em diferentes cenários de atuação profissional pública ou privada. Ante o exposto, formulou-se a seguinte questão norteadora: quais as experiências de homens transexuais durante o parto e pós-parto? Para tanto, objetivou-se desvelar as experiências de homens transexuais durante o parto e pós-parto à luz da Teoria da Diversidade e Universalidade do Cuidado Cultural. 

## Método

### Delineamento e local do estudo

 Trata-se de um estudo qualitativo ^(^ 9 ^-^ 10 ^)^ e operacionalizado pelo método de Estudo de Casos Múltiplos ^(^ 11 ^)^ . O cenário investigado é vinculado à Secretaria Estadual de Saúde, por meio da Coordenação Estadual de Atenção Integral à Saúde Lésbicas, Gays, Bissexuais, Travestis e Transexuais (LGBT) de Pernambuco (PE), e em nível nacional com pessoas vinculadas à Aliança Nacional LGBT. 

### Definição da amostra

 Participaram do estudo cinco homens transexuais “parturientes”. A amostragem foi do tipo intencional ^(^ 12 ^)^ , composta por participantes selecionados a partir de critérios de conveniência e disponibilidade, após estabelecidos os seguintes critérios de inclusão: ser homem transexual com vivência em trabalho de parto vaginal ou cesárea; ter idade igual ou superior aos 18 anos de idade. Os critérios de exclusão foram: homens transexuais que vivenciaram o trabalho de parto e que não possuíam recursos tecnológicos como computador, celular, *Internet,* entre outros, para possibilitar a realização da entrevista. Foram incluídos os participantes por inserção progressiva, a partir da saturação teórica das respostas às entrevistas semiestruturadas ^(^ 13 ^)^ . 

### Coleta de dados

 A coleta de dados foi realizada nos meses de setembro e outubro de 2021. Para tanto, foi utilizada a técnica de entrevistas individuais ^(^ 13 ^)^ , em modalidade virtual (remota), pela plataforma *Google Meet* ^®^ , em encontro único, com roteiro semiestruturado, composto por questões fechadas relacionadas às características sociodemográficas e de questões abertas relacionadas ao fenômeno empírico investigado, a saber: 1. Fale-me sobre a sua experiência durante o momento do parto e pós-parto; 2. Conte-me como ocorreu a assistência à saúde prestada pela equipe de saúde durante o seu parto e o pós-parto; 3. Caso você tenha optado por amamentar no pós-parto, fale-me como se deu esta experiência; 4. Você conhece outros homens transexuais que engravidaram ou que estejam grávidos? Conte-me sobre as experiências que conheceu. A caracterização sociodemográfica dos participantes, foi realizada após a disponibilização um questionário antes das entrevistas. Durante a captação dos participantes a partir do contato com as instituições parceiras, que ocorreu nas principais redes sociais digitais acessadas no Brasil: *Facebook* ^®^ , *Instagram* ^®^ e *WhatsApp* ^®(14)^ . 

 As anuências para o Termo de Consentimento Livre e Esclarecido (TCLE) e o Termo de Autorização de Uso de Imagem e Depoimento foram disponibilizadas para a assinatura pela plataforma *Google Forms* ^®^ ( *online* ). Para cada entrevista foi solicitado ligar a câmera do celular ou do computador para a captação de imagem, linguagem corporal e entonações de voz pela plataforma *Google Meet* ^®^ , obtendo-se um tempo médio de uma hora para cada uma delas. 

 As entrevistas foram realizadas pelo pesquisador principal, que é integrante da comunidade LGBT e possui *expertise* na execução de estudos qualitativos, o que contribuiu para o processo de coleta das informações, que foram posteriormente transcritas na íntegra e disponibilizadas aos participantes para que fosse realizada a validação do conteúdo, realizando ajustes e correções, se necessário. Nessa etapa, todos os participantes leram o material e não foram sugeridas mudanças no conteúdo. Todo o material foi submetido à análise lexicográfica clássica por meio do Método Reinert ^(^ 15 ^)^ de classificação de segmentos de texto instrumentalizada pelo *software* gratuito *Interface de R pour les Analyses Multidimensionnelles de Textes et de Questionnaires* (IRAMuTeQ) versão 7.0 ^(^ 16 ^)^ . A partir do emprego do IRAMuTeQ foi possível categorizar os dados, mediante a avaliação de similaridade ^(^ 17 ^-^ 19 ^)^ . 

### Tratamento e análise dos dados

 Os achados centrais encontrados no Estudo de Casos Múltiplos realizado, foram discutidos teoricamente a partir dos pressupostos da TDUCC ^(^ 20 ^)^ . A análise dos dados foi ancorada na TDUCC, proposta por Madeleine Leininger, em que se aborda a necessidade de o profissional considerar o contexto cultural no qual o indivíduo se encontra inserido, propiciando cuidados harmônicos àquela identidade local, sendo elencados cinco conceitos que a sustentam, sendo eles a cultura, visão de mundo, contexto ambiental, cuidado e saúde; associado à literatura científica pertinente à temática ^(^ 20 ^)^ . 

### Aspectos éticos

 Este estudo foi submetido à aprovação pelo Comitê de Ética em Pesquisa da Universidade Federal de Pernambuco (UFPE), sob o CAAE nº 47777421.0.0000.5208 e parecer de número 4.862.503/2021, seguindo as recomendações da Resolução nº 466, de 12 de dezembro de 2012 do Conselho Nacional de Saúde brasileiro ^(^ 21 ^)^ . 

## Resultados

 Dispõem-se na Figura 1 os casos múltiplos dos homens transexuais “parturientes” do estudo, identificados por pseudônimos autodeclarados no momento das entrevistas, de acordo com o perfil gravidez-parto. 

**Figura 1:** - Casos múltiplos dos homens transexuais participantes do estudo (n = 5). Recife, PE, Brasil, 2023

 Na Figura 2 apresenta-se o agrupamento dos registros mais significativos por conceito elencado a partir da adaptação do Modelo *Sunrise* , proposto pela TDUCC, alocando as falas em consonância com as experiências dos homens transexuais durante o parto e pós-parto. Assim, os relatos expõem o medo do parto, a violência obstétrica, transfóbica, moral, psicológica, sexual ou por negligência, entre outros, conforme apresentado abaixo. 

**Figura 2:** - Experiências atribuídas ao parto e pós-parto imediato por homens transexuais, adaptadas do Modelo *Sunrise* . Recife, PE, Brasil, 2023

 Na Figura 3 tem-se o resultado da construção da adaptação do Modelo *Sunrise,* traduzida em ideias-chaves mais recorrentes nos discursos dos homens transexuais parturientes, evidenciando os aspectos intrínsecos ao parto e pós-parto vivenciados por estes sujeitos, a serem considerados para o reconhecimento de um cuidado transcultural. Na Figura 4 evidencia-se uma relação entre os fatores culturais e os elementos presentes nos discursos dos indivíduos participantes do estudo. 

**Figura 3:** - Adaptação do Modelo *Sunrise* . Valores que influenciam os cuidados de saúde aos parturientes. Recife, PE, Brasil, 2023

**Figura 4:** - Fatores culturais que influenciam os cuidados de saúde e os elementos presentes na fala dos parturientes. Recife, PE, Brasil, 2023

## Discussão

Destaca-se a incipiência de literatura científica nacional e internacional que abordem as especificidades de homens transexuais durante a gestação, parto e puerpério, reafirmando a necessidade deste estudo, que engloba a Obstetrícia e a Enfermagem nas discussões sobre as relações entre saúde, gênero e sexualidades. Ante o exposto, este estudo apresenta relevantes contribuições e implicações nestas áreas e no avanço do conhecimento científico na assistência pré-natal e obstétrica para as diferentes identidades de gênero, sobretudo quanto à visibilidade das experiências de homens transexuais durante o parto e o pós-parto imediato, sob a análise dos conceitos de “Cultura”, “Visão de mundo”, “Contexto ambiental”, “Cuidado” e “Saúde” proposto pela TDUCC de Madeleine Leininger.

 No item referente a “cultura”, registrou-se a percepção de que os homens transexuais tinham em relação ao parto natural e aos atos de violência ocorridos nesse momento, por vezes naturalizados. Nesse sentido, o estigma social que culturalmente considera o parto normal como perigoso, que gera dor e sofrimento, exerce uma influência significativa sobre as experiências de homens transexuais. Este aspecto corrobora o que Leininger afirmou, ou seja, que a cultura poderá guiar o indivíduo ou um grupo em suas ações e construir a maneira como gostaria de ser cuidado. Parte desse sentimento relaciona-se à assistência de saúde ofertada pelo Sistema Único de Saúde (SUS), muitas vezes vista como desqualificada, uma vez que acredita-se que os atendimentos ocorrem após longos períodos de espera, e justifica-se, com isso, a escolha pela cirurgia cesárea mesmo diante de condições para realizar o parto natural, relacionando a via cirúrgica como rápida e segura ^(^ 22 ^)^ . 

 Destaca-se que, com a medicalização do parto, difundiu-se socialmente que os efeitos de remédios e procedimentos técnicos e tecnológicos realizados por profissionais da saúde são mais eficientes que a própria fisiologia corporal. As evidências científicas relatam que os principais fatores que desencadeiam o medo do parto natural entre os homens transexuais são os relatos de dor por familiares e amigos que passaram por esse momento, a ansiedade relacionada ao sofrimento durante o parto normal, a insegurança de não ser capaz de concluir o parto e o temor que ele cause complicações ao recém-nascido (RN) ^(^ 23 ^)^ . O medo deste momento pode estar relacionado às mudanças, também, do canal vaginal (a parede da vagina consiste em três camadas: mucosa, muscular e adventícia), devido ao uso de testosterona durante o processo de afirmação de gênero e as preocupações sobre os cuidados insuficientes, como a falta de recursos em serviços de saúde, ou relativas à competência transcultural dos profissionais em assistir o parto de um homem transexual, visto a incipiência desta discussão nos cursos de formação em saúde ^(^ 3 ^-^ 4 ^,^ 24 ^)^ . 

 Em uma das experiências de um dos participantes do estudo, ao qual “o parturiente” optou pela via de parto normal, ocorreram os procedimentos de indução do parto e da episiotomia. Observou-se a ausência de informação por parte do usuário sobre do que se tratava e a tomada de decisão na realização dos procedimentos sem o seu devido consentimento explícito e informado, o que pode se caracterizar também como uma violência obstétrica. Destaca-se que, apesar dos critérios para realizá-la, a indução é considerada um procedimento invasivo, e que pode impactar na experiência do parto e no nascimento, aumentando o tempo de espera e até implicando em complicações obstétricas, a exemplo do risco de laceração perineal e hemorragia pós-parto ^(^ 25 ^)^ . 

 Um “dos parturientes” que teve como via de parto a cesárea eletiva afirmou ter de permanecer isolado no pós-parto imediato, tornando este um momento solitário. O sentimento que perpassou esta vivência assemelhou-se ao que pode ser denominado de “solidão puerperal”, caracterizada pela exacerbação da solidão após o parto, devido à impossibilidade de visitas ou à extensão do internamento dele ou do RN ^(^ 26 ^)^ . Ainda assim, a cesárea mostrou-se uma opção viável para os homens transexuais que vivenciaram a gestação, sendo a via de parto escolhida por quatro dos cinco “parturientes”. 

As relações que homens transexuais estabelecem junto aos profissionais da saúde, especialmente entre os que o acompanham durante a assistência ao parto e ao pós-parto imediato, influenciaram de maneira significativa o cuidado prestado. Verifica-se a reprodução de uma deslegitimação da identidade de gênero “do parturiente”, a partir de sua identificação pelo gênero feminino na etiqueta do RN na sala de parto, o que gerou um momento de intenso de estresse, de forma que esta situação se caracteriza como uma violência transfóbica, o que demonstra o despreparo na formação profissional e da instituição de saúde.

 Estas situações podem ocorrer, ainda, no momento do registro do RN, ainda no serviço de saúde. Assim, no Brasil, o Supremo Tribunal Federal (STF) determinou que o Ministério da Saúde, a partir do mês de setembro do ano de 2021, realizasse alterações na Declaração de Nascido Vivo (DNV) e no Sistema de Informações de Nascidos Vivos (SINASC), para que se faça constar neste documento a categoria “parturiente”, independente dos nomes dos genitores. Isso possibilita o recolhimento de dados para a formulação de políticas públicas e o respeito à autodeclaração de gênero dos ascendentes ^(^ 27 ^)^ . 

 Verifica-se, também, que após o aumento do uso de técnicas de fertilização *in vitro,* adoções e demais recursos para garantir a parentalidade entre a população LGBT, especialmente entre homens transexuais, a certidão de nascimento passou a não fazer referência aos genitores como “mãe” e “pai”, utilizando-se do termo “filiação” a partir de 2017, com o Provimento nº 63 do Conselho Nacional de Justiça (CNJ) ^(^ 28 ^-^ 29 ^)^ . 

O contexto ambiental apresentou-se no relato de afastamento da criança “do parturiente” após um parto cesáreo, bem como a ocorrência de uma situação de violência sexual quando o usuário se encontrava sob os efeitos da anestesia. Estas situações se somam às diversas experiências negativas que ocorrem no âmbito hospitalar, com reflexos no sofrimento e no gatilho de adoecimento mental das pessoas transgênero.

Este ambiente que se faz presente no momento do parto e pós-parto imediato pode ser decisivo sobre quais as impressões a pessoa irá atribuir à sua experiência. Nesse sentido, a violência sexual sofrida por um “dos parturientes” se fez presente, e este verbalizou a ocorrência de uma “molestação” por parte de um profissional da saúde, momentos após o parto cesáreo, em uma tentativa de se aproveitar dos efeitos da raquianestesia para praticar a ação criminosa. Nessa situação, após denunciar a violência sofrida para a genitora que o acompanhava no serviço de saúde, “o parturiente” sofreu mais uma violência quando não foi ouvido, tendo sua experiência posta em questionamento, uma vez que estava sob os efeitos de medicamentos.

A centralidade do cuidado esteve na prática do uso de técnicas não-farmacológicas para o enfrentamento às dores do parto, ao mesmo tempo que se observa o desrespeito “ao parturiente” por parte da profissional da saúde, tornando a assistência fragilizada e violenta. O uso de formas não farmacológicas de alívio da dor durante o trabalho de parto, a exemplo da musicoterapia, massoterapia, termoterapia e uso da bola suíça, apareceram como estratégias utilizadas pelos profissionais da saúde, de maneira a possibilitar um apoio para que as pessoas parturientes pudessem enfrentar as dores do trabalho de parto, uma vez que este é um evento visto como uma das experiências mais significativas para as pessoas envolvidas.

A categoria “saúde” expressou o sentimento “do parturiente” sobre o serviço de saúde prestado durante o parto, observando-se a insatisfação da assistência proporcionada pela instituição, ao mesmo tempo que ele reconhece o cuidado da profissional da saúde em oferecer um acolhimento e apoio ao enfrentamento das situações de violência presentes naquele momento. Isso gerou um sentimento de esperança no usuário. Observou-se, também, a importância do papel do profissional da saúde em fazer com que “o parturiente” o visse como um apoio no compartilhamento de suas angústias, estabelecendo um vínculo positivo e gerando sentimentos de apoio e confiança no enfrentamento das situações de violência. Este fato evidencia a necessidade de se considerarem as especificidades da pessoa parturiente durante o cuidado obstétrico, a partir do reconhecimento e da valorização da sua singularidade, de sua cultura e visão de mundo.

 A categoria “visão de mundo” relaciona-se com o processo de amamentação, revelando-se como um fenômeno que constitui a masculinidade dos sujeitos e é possível de ser realizada no pós-parto imediato, objetivando a manutenção da alimentação da criança neste momento da vida. Quanto à opção ou não de homens transexuais “parturientes” amamentarem, verifica-se que os participantes deste estudo se dispuseram a realizá-la no pós-parto imediato, e por vezes ocorreu o estímulo à amamentação pela equipe de saúde na primeira hora de vida do RN. Em um dos “parturientes”, que estava em uma relação estável transcentrada heterossexual, o aleitamento ofertado à criança ocorreu por meio da companheira, uma mulher transexual, para que esta pudesse cumprir com o papel social de “mãe’ tornando-se a responsável por este processo até o quarto mês de vida do RN, sendo iniciada em seguida a introdução de fórmula láctea infantil e o retorno do uso da testosterona pelo homem transexual ^(^ 30 ^)^ . 

 Ressalta-se a importância do papel do enfermeiro no processo de educação em saúde sobre as orientações dos riscos e dos benefícios da amamentação para os homens transexuais parturientes que não realizaram o procedimento cirúrgico da mastectomia masculinizadora, durante toda a assistência pré-natal, para que possam refletir e tenham a autonomia de decidir se irão ou não realiza-la, compreendendo este acontecimento como constituinte de sua masculinidade. Para isso, espaços como o alojamento conjunto tornam-se propícios para a realização deste tipo de orientação pelo enfermeiro ^(^ 31 ^)^ . 

 O cuidado obstétrico-neonatal, frequentemente marcado pela violação de direitos e pela cultura cis-heteronormativa, mostra que, além de lidar com o trabalho de parto, um momento já desafiador, os homens transexuais gestantes também lidam com o preconceito e a discriminação, que podem ser caracterizadas como violência obstétrica, uma vez que oferecem danos físicos ou psicológicos “ao parturiente” ^(^ 32 ^)^ . 

 Informa-se que este estudo apresentou limitações relacionadas ao número de participantes, por se tratar de uma população específica e de difícil acesso, agravando-se este durante o período da pandemia da COVID-19, diante de um contexto de vulnerabilidade socioeconômica. Diante de uma amostra por conveniência, ocorreram também, alguns impasses na inserção de homens transexuais no estudo, pois alguns deles não retornavam o contato com o pesquisador ou não apresentavam interesse em participar da pesquisa realizada por uma pessoa cisgênero. Além disso, os achados evidenciam o debate recente no Brasil e no mundo sobre as transparentalidades, bem como ampliam o escopo da atenção à saúde dos homens, considerando as distintas identidades de gênero, como no caso dos homens trans e suas demandas de saúde sexual, reprodutiva e familiar ^(^ 33 ^-^ 36 ^)^ . 

Com o intuito de minimizar as possíveis limitações metodológicas no decorrer da pesquisa, foram estritamente seguidas as etapas de aproximação e seleção dos participantes. Uma divulgação abrangente do estudo foi realizada para evitar viés na estratégia de recrutamento. As técnicas utilizadas para apreender as experiências de homens transexuais durante o parto e pós-parto foram aplicadas de maneira coerente, e o referencial teórico foi empregado de forma responsável. Adicionalmente, foram conduzidas as atividades preliminares para a construção do estudo, visando reforçar a sensibilidade teórica do pesquisador principal e da equipe. Isso incluiu a integração dos participantes nos contextos relevantes, investimento em leituras, participação em disciplinas curriculares e capacitações específicas na área.

## Conclusão

Este estudo evidenciou que o estigma social que culturalmente enfatiza o parto normal como perigoso, que gera dor e sofrimento, exerceu influência significativa na escolha da via de parto entre os homens transexuais, reforçando que as experiências vivenciadas são acometidas por diferentes situações de violência, seja moral, psicológica, sexual, transfóbica ou por negligência. A visão de mundo “do parturiente” leva à necessidade de atenção hospitalar que reconheça sua singularidade. Já o contexto ambiental pode revelar maior vulnerabilidade para a ocorrência de situações que possam gerar impactos negativos na assistência ao parto e pós-parto. O cuidado mostra-se potencializado a partir da realização de estratégias no apoio ao enfrentamento às situações intrínsecas ao trabalho de parto e à saúde, porém mediante as violências e a violação de direitos torna-se fragilizado.
